# Fractional Anisotropy and Troponin T Parallel Structural Nerve Damage at the Upper Extremities in a Group of Patients With Prediabetes and Type 2 Diabetes – A Study Using 3T Magnetic Resonance Neurography

**DOI:** 10.3389/fnins.2021.741494

**Published:** 2022-01-24

**Authors:** Johann M. E. Jende, Zoltan Kender, Jakob Morgenstern, Pascal Renn, Christoph Mooshage, Alexander Juerchott, Stefan Kopf, Peter P. Nawroth, Martin Bendszus, Felix T. Kurz

**Affiliations:** ^1^Department of Neuroradiology, Heidelberg University Hospital, Heidelberg, Germany; ^2^Department of Endocrinology, Diabetology and Clinical Chemistry, Heidelberg University Hospital, Heidelberg, Germany; ^3^German Center of Diabetes Research, München, Germany; ^4^Joint Institute for Diabetes and Cancer at Helmholtz-Zentrum Munich and Heidelberg University, Heidelberg, Germany; ^5^German Cancer Research Center, Radiology E010, Heidelberg, Germany

**Keywords:** diabetic polyneuropathy, magnetic resonance neurography (MRN), diffusion tensor imaging, microangiopathy, high-sensitivity Troponin T

## Abstract

**Background:**

Recent studies have found that troponin T parallels the structural and functional decay of peripheral nerves at the level of the lower limbs in patients with type 2 diabetes (T2D). The aim of this study was to determine whether this finding can also be reproduced at the level of the upper limbs.

**Methods:**

Ten patients with fasting glucose levels >100 mg/dl (five with prediabetes and five with T2D) underwent magnetic resonance neurography of the right upper arm comprising T2-weighted and diffusion weighted sequences. The fractional anisotropy (FA), an indicator for the structural integrity of peripheral nerves, was calculated in an automated approach for the median, ulnar, and radial nerve. All participants underwent additional clinical, serological, and electrophysiological assessments.

**Results:**

High sensitivity Troponin T (hsTNT) and HbA1c were negatively correlated with the average FA of the median, ulnar and radial nerve (*r* = −0.84; *p* = 0.002 and *r* = −0.68; *p* = 0.032). Both FA and hsTNT further showed correlations with items of the Michigan Hand Outcome Questionnaire (*r* = −0.76; *p* = 0.010 and *r* = 0.87; *p* = 0.001, respectively). A negative correlation was found for hsTNT and HbA1c with the total Purdue Pegboard Test Score (*r* = −0.87; *p* = 0.001 and *r* = −0.68; *p* = 0.031).

**Conclusion:**

This study is the first to find that hsTNT and HbA1c are associated with functional and structural parameters of the nerves at the level of the upper limbs in patients with impaired glucose tolerance and T2D. Our results support the hypothesis that hyperglycemia-related microangiopathy, represented by elevated hsTNT levels, is a contributor to nerve damage in diabetic polyneuropathy.

## Introduction

Diabetic neuropathy (DN) is one of the most prevalent and yet most poorly understood complications of diabetes mellitus resulting in a reduction of affected patients’ quality of life and an enormous economic burden for the global healthcare system (Davies et al., 2006; Alleman et al., 2015). Although several pathophysiological pathways associated with DN have been identified and investigated in both preclinical and clinical studies, clinical studies on reliable markers that allow for both quantification and prediction of nerve damage in DN have come to controversial results (Callaghan et al., 2012; Spallone and Greco, 2013; Feldman et al., 2017).

In addition, it is well known that upper and lower limb nerves in DN behave differently regarding their functional properties and demands, which may result from the influence of length-dependent factors on neurological development, see e.g., Dyck et al. (1985). More recent works also indicate upper and lower limb specific nerve conduction velocities (NCVs) in contrast to their nerve cross-section area development (Nobue et al., 2020), and it has also been argued that a differential expression of slow hyperpolarization-activated cyclic nucleotide-gated channel isoforms between upper and lower limbs may contribute to earlier dysfunction of lower limbs in neuropathy (Marmoy et al., 2019). However, while DN is generally agreed to be a length dependent neuropathy, both histological studies (Dyck et al., 1986a) and recent imaging studies have found that structural nerve damage linked to demyelination in affected patients predominates at a proximal level of the affected extremities in patients with diabetes and prediabetes (Pham et al., 2015; Jende et al., 2018, 2021), indicating that the entire peripheral nervous system is affected throughout the entire course of DN and that, even in prediabetes, when blood glucose levels are at a lower level than in type 2 diabetes (T2D), structural nerve damage at a proximal level can already be detected (Jende et al., 2021).

One of the most challenging obstacles and potential confounder for clinical studies on DN of the upper extremities is that clinical examinations, such as clinical scores, and electrophysiological examinations are dependent on the patient’s cooperation and individual perception of symptoms, as well as the rater’s experience. In addition, electrophysiological examinations can be perceived as unpleasant or even painful. They also do not allow any conclusions on potential anatomical location, size, distribution, or composition of nerve lesions. Studies on non-invasive in vivo high resolution magnetic resonance neurography (MRN) have shown that diffusion tensor imaging (DTI) allows a very accurate and reproducible assessment of a nerve’s structural integrity, thus providing a more objective tool for the assessment of nerve damage than clinical examinations alone, see e.g., Kronlage et al. (2018), or Jeon et al. (2018) and references therein. For instance, Lehmann et al. (2010) used DTI to reliably assess axonal regeneration in an animal model of crush neuropathy using high-field MRI at 11.7 Tesla. Specifically in the context of polyneuropathy, recent studies could show that DTI can be used to visualize, detect, and quantify neuropathic changes in human type 1 and type 2 diabetes (T2D) in the lower leg (Vaeggemose et al., 2017, 2020; Edward et al., 2020; Xia et al., 2021).

In addition, a recent study on diffusion-weighted MRN found that elevated levels of Troponin T obtained from high sensitivity Troponin T (hsTNT) assays are associated with structural nerve damage at the level of the lower limbs in patients with T2D, supporting the hypothesis that hsTNT is a potential serological marker for microvascular disease and associated pathologies in T2D (Jende et al., 2020a). This is in line with previous studies that had shown hsTNT levels to be associated with the occurrence of microvascular complications in patients with T2D (Li et al., 2014). Also, it could be shown that the structural integrity of the sciatic nerve in patients with diabetes and prediabetes is related to the functionality of patient’s arms and hands (Jende et al., 2020b,^2021^).

The rationale for this study was therefore to examine nerve integrity in upper arm nerves in prediabetes and diabetes and its correlation to functional (clinical and electrophysiological) scores and specific serological markers of nerve ischemia and glucose control, to test, validate, and compare with earlier findings in lower limbs. Specifically, we investigated whether hsTNT parallels structural nerve damage and a decline in nerve function at the level of the upper limbs.

## Materials and Methods

### Study Design and Participants

The local ethics committee (HEIST-DiC, local ethics number S-383/2016, clinicaltrials.gov identifier NCT03022721) approved this study and all participants gave written informed consent. Twelve patients with impaired glucose tolerance (six with prediabetes and six with T2D) took part in this prospective study between June 2018 and June 2019. Ten participants completed the study protocol (five with prediabetes and five with T2D; three female, seven male, mean age 59.30 years ± 9.36; age range 42–75 years). Overall exclusion criteria were age <18, pregnancy, any contraindications for MR imaging, any history of cervical surgery or disc extrusion, any other risk factors for neuropathy such as alcoholism, malignant or infectious diseases, hypovitaminosis, monoclonal gammopathy, any previous or ongoing exposure to neurotoxic agents, and any chronic neurological diseases such as Parkinson’s disease, restless leg syndrome, or multiple sclerosis. Additional exclusion criteria for controls were any sings of neuropathy in the participants medical history or in the clinical and electrophysiologic examinations as shown below.

### Clinical and Electrophysiological Examination

For every patient, a detailed medical history was documented and a physical examination including the neuropathy disability score (Young et al., 1993) was performed. Since no standardized scoring system for the characterization of DN at the level of the upper extremities has been established to date, the overall presence of DN was determined using the well-known Gibbon’s criteria according to which patients with an NDS ≥ 3 had DN, and no DN for NDS < 3 (Gibbons et al., 2010). The electrophysiological examination (Viasys Healthcare VikingQuest^®^, Viasys Healthcare GmbH, Höchberg) of the right arm included: distal motor latencies of the right median and ulnar nerve, motor and sensory amplitudes [compound muscle action potential (CMAPs) and sensory nerve action potential (SNAPs), respectively] of the median and ulnar nerve and both motor and sensory NCVs of the median and ulnar nerve. It was assured that skin temperature was at least 32°C throughout the examination. Since there is no established protocol for the evaluation of hand function in patients with diabetes and prediabetes, we chose the well-established Purdue-Pegboard score for the assessment of motor hand function in general (Tiffin and Asher, 1948) and items of daily life activity from the Michigan Hand Outcome Questionnaire (MHOQ; Shauver and Chung, 2013). Blood was drawn in fasting state and processed immediately under standardized conditions in the Central Laboratory of the University Hospital of Heidelberg. Albumin excretion in urine was detected in morning spot urine within all participants. Estimated glomerular filtration rate (GFR) was calculated with the CKD-EPI-formula (Levey et al., 2009). Prediabetes was defined as fasting glucose levels of 100–125 md/dL, and T2D was defined as fasting glucose levels of >125 md/dL. The serological parameter hsTNT was determined on Cobas E411 (Roche Diagnostics Ltd., Rotkreuz, Switzerland) using heparin plasma. As in previous works (Giannitsis et al., 2010), the limit of blank was 3 ng/L, and the limit of detection was 5 ng/L; both were found in compliance with guideline EP17-A of the Clinical and Laboratory Standards Institute. Further details on analytical characteristics and assay performance can be found in Giannitsis et al. (2010).

### Magnetic Resonance Neurography Imaging Protocol

All participants underwent high-resolution MRN of the right upper arm in a 3.0 Tesla MR-scanner (Magnetom SKYRA, Siemens Healthineers, Erlangen, Germany). Participants were examined in prone position with the arm in elbow extension, and upper arm placement within the extremity coil was aligned with the longitudinal axis of the upper arm at an angle of 10° to the external magnetic induction field B_0_ of the MR-scanner to avoid magic angle effects. A 15-channel transmit-receive extremity coil was employed and the following sequences were applied:

(1) Axial high-resolution T2-weighted turbo spin echo (TSE) 2D sequence with spectral fat saturation (mode: strong) without water suppression or magnetization preparation. Repetition time (TR) = 5,970 ms, echo time (TE) = 55 ms, field of view (FOV) = 160 mm^2^ × 160 mm^2^, matrix size = 512 × 512, slice thickness = 4 mm, interslice gap = 0.8 mm, voxel size = 0.3 mm^3^ × 0.3 mm^3^ × 4.0 mm^3^, 24 slices, 24 acquired images, receiver bandwidth = 181 Hz/pixel, echo spacing = 11.1 ms, turbo factor = 13, 15 echo trains per slice, parallel imaging factor = 2, averages = 3, acquisition time = 4:42 min.

(2) Axial diffusion-weighted two-dimensional echo-planar sequence images were recorded with spectral attenuated inversion recovery fat suppression (saturation mode: skewed): TR = 5100 ms; TE = 92.8 ms; *b* = 0 and 1000 s/mm^2^; directions = 20; FOV = 160 mm^2^ × 160 mm^2^; matrix size = 128 × 128; slice thickness = 4 mm; voxel size = 1.3 mm × 1.3 mm × 4 mm; no interslice gap, 24 slices, 1512 acquired images, receiver bandwidth = 1,396 Hz/pixel, EPI factor = 128, parallel imaging factor = 3, averages = 3, acquisition time = 5:47 min.

In each participant, the sequence was centered to the middle of the upper arm.

### Image Post-processing and Statistical Analysis

**FIGURE 1:**
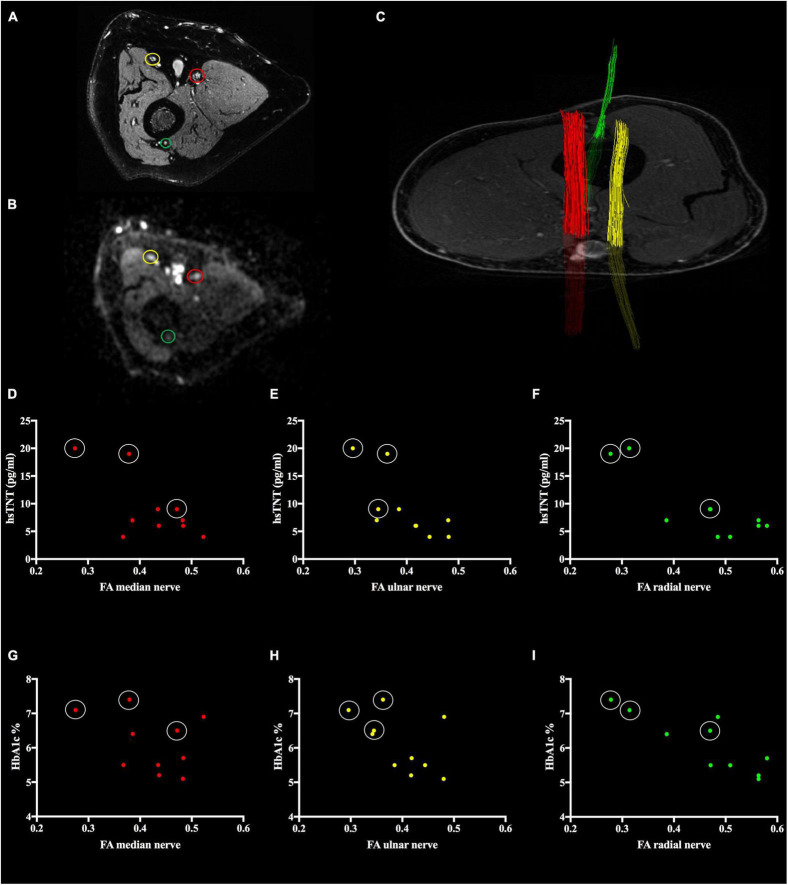
Segmentation of nerve fiber tracts from diffusion tensor imaging and correlation of nerves’ fractional anisotropy (FA) with high sensitivity Troponin T (hsTNT) assays. **(A)** T2-weighted sequence of the right upper arm. The median (red circle), ulnar (yellow circle), and radial (green circle) nerve are visible. **(B)** Diffusion-weighted image at *b* = 1000 s/mm^2^ of the same region as is **A**. **(C)** Three-dimensional reconstruction of the median (red), ulnar (yellow), and radial (green) nerve. **(D)** hsTNT vs median nerve’s FA (*r* = –0.67; *p* = 0.069), **(E)** hsTNT vs ulnar nerve’s FA (*r* = –0.74; *p* = 0.035), **(F)** hsTNT vs radial nerve’s FA (*r* = –0.86; *p* = 0.007), **(G)** HbA1c vs median nerve’s FA (*r* = –0.34; *p* = 0.334), **(H)** HbA1c vs ulnar nerve’s FA (*r* = –0.53; *p* = 0.113), **(I)** HbA1c vs radial nerve’s FA (*r* = –0.84; *p* = 0.002). In **D–I**, values of patients with diabetic neuropathy are encircled in white.

All images generated were pseudonymized. Images were analyzed in an automated approach using Nordic BRAINEX, a clinical software designed for automated calculation and reconstruction of fiber tracks in diffusion weighted imaging (O’Donnell et al., 2017). T2-weighted and diffusion-weighted sequences were coregistered automatically and the region of the median, ulnar and radial nerve was marked by a trained neuroradiologist with 6 years of experience in MRN imaging (Figures 1A–C). Based on the results of former studies on DTI in the sciatic nerve, the nerve’s fiber tracks were automatically segmented with a threshold of >0.1 for the nerve’s fractional anisotropy (FA), a dimensionless quantity for directed diffusion that has been shown to be correlated with axonal and myelin integrity (Kronlage et al., 2018; Jende et al., 2021). The average FA was calculated as the arithmetic mean of the radial, ulnar and median nerve’s FA.

### Statistical Analysis

Statistical data analysis was performed with GraphPad Prism 6. All data were tested for Gaussian normal distribution using the D’Agostino-Pearson omnibus normality test. Also, we applied the iterative Grubbs’ test to identify and exclude potential outliers. If a Gaussian normal distribution was given, Pearson correlation coefficients were calculated for correlation analysis. If data were not Gaussian distributed, non-parametric Spearman correlation was applied for correlation analysis. For all tests, the level of significance was defined at *p* < 0.05. All results are presented as mean values ± standard deviation (SD).

## Results

### Demographic and Clinical Data

Ten participants completed the MRI scans. Of those, three had DN and seven had no DN. Grubbs test identified 3 electrophysiological values as outliers, which were removed for analysis. No outliers were identified for serological or MRN imaging data. A summary of all demographical, clinical, electrophysiological, serological, and MRN parameters acquired is provided in Table 1.

**TABLE 1:** Demographic, clinical, serological, and MRN imaging data of all study participants.

Female	3
Male	7
Age (years)	59.30 ± 9.36
Body-mass-index (kg/m^2^)	2934 ± 6.10
Median nerve fractional anisotropy	0.42 ± 0.07
Ulnar nerve fractional anisotropy	0.40 ± 0.06
Radial nerve fractional anisotropy	0.46 ± 0.10
Average fractional anisotropy	0.43 ± 0.07
Total Purdue Pegboard Test Score	63.0 ± 18.83
Daily life items of the Michigan Hand Outcome Questionnaire	3.90 ± 5.45
Median nerve motor conduction velocity (m/s)	50.89 ± 3.59
Median nerve CMAP (μV)	9.08 ± 3.19
Median nerve distal motor latency (ms)	3.60 ± 0.28
Median nerve sensory conduction velocity (m/s)	52.25 ± 7.38
Median nerve SNAP (μV)	12.49 ± 5.65
Ulnar nerve motor conduction velocity (m/s)	54.56 ± 4.39
Ulnar nerve CMAP (μV)	10.34 ± 2.43
Ulnar nerve distal motor latency (ms)	2.69 ± 0.31
Ulnar nerve sensory conduction velocity (m/s)	52.88 ± 7.38
Ulnar nerve SNAP (μV)	10.59 ± 5.26
Skin auto-fluorescence	2.32 ± 0.49
High sensitivity Troponin T (pg/mL)	9.10 ± 5.7
proBNP (pg/mL)	53.11 ± 26.67
HbA1c (%)	6.13 ± 0.83
Glomerular filtration rate (mL/min)	92.02 ± 13.74

*All values are displayed as mean ± SD. proBNP: pro brain natriuretic peptide. m/s, meters per second; ms, milliseconds; μV, microvolts; pg/mL, picogram per milliliter; mL/min, milliliters per minute.*

### Correlation of Magnetic Resonance Neurography Data With Demographic, Clinical, and Serological Data

The average FA of the nerves at the upper right arm was negatively correlated with hsTNT and HbA1c (*r* = −0.84; *p* = 0.002 and *r* = −0.68; *p* = 0.031). In a controlled regression analysis, the correlation between hsTNT and FA remained significant when controlled for HbA1c (*r* = 0.71; *p* = 0.029), whereas the correlation between FA and HbA1c levels did not remain significant when controlled for hsTNT (*r* = −0.31; *p* = 0.417). Individual correlations of the median, ulnar, and radial nerves are displayed in Figures 1D–I, in Table 2, and in Supplementary Table 1. Further negative correlations of the average FA were found for skin auto-fluorescence (−0.68; *p* = 0.031) and the assessed items of the MHOQ (*r* = −0.76; *p* = 0.010). Positive correlations were found with the median nerve SNAP (*r* = 0.80; *p* = 0.029) and the ulnar nerve SNAP (*r* = 0.89; *p* = 0.001). No correlations were found with age, body-mass-index (BMI), or GFR.

**TABLE 2:** Correlation of HbA1c, high sensitivity Troponin T, and fractional anisotropy of the nerves of the upper limb with demographic, clinical, and serological data.

	HbA1c		hsTNT (pg/ml)		Average FA		Median nerve FA		Ulnar nerve FA		Radial nerve FA	
	*r*	*p*	*r*	*p*	*r*	*p*	*r*	*p*	*r*	*P*	*r*	*p*
Average FA	–0.68	0.031	–0.84	0.002			0.87	0.001	0.87	0.001	0.93	<0.001
Median nerve FA	–0.34	0.334	–0.68	0.032	0.87	0.001			0.69	0.026	0.69	0.027
Ulnar nerve FA	–0.53	0.113	–0.72	0.019	0.87	0.001	0.69	0.026			0.72	0.018
Radial nerve FA	–0.84	0.002	–0.83	0.003	0.93	<0.001	0.69	0.027	0.72	0.018		
Age (years)	0.44	0.200	0.39	0.271	–0.35	0.316	–0.12	0.732	–0.45	0.189	–0.37	0.289
BMI (kg/m^2^)	0.17	0.635	–0.03	0.931	0.1	0.777	0.24	0.513	0.14	0.697	–0.04	0.921
Total Pegboard score	–0.68	0.031	–0.87	0.001	0.63	0.05	0.43	0.219	0.6	0.065	0.64	0.044
Daily life items of the MHOQ	0.59	0.075	0.87	0.001	–0.76	0.01	–0.73	0.018	–0.61	0.061	–0.70	0.024
Skin auto-flourescence	0.75	0.012	0.9	<0.01	–0.68	0.031	–0.47	0.166	–0.5	0.142	–0.77	0.009
proBNP (pg/mL)	0.16	0.689	0.28	0.47	–0.23	0.552	0.06	0.869	–0.37	0.327	–0.33	0.392
GFR (mL/min)	0.39	0.265	0.15	0.689	<0.01	0.994	0.10	0.774	0.31	0.389	–0.26	0.47

### Correlation of Troponin T and HbA1c With Clinical Data

Parameter hsTNT showed negative correlations with the total Pegboard score (*r* = −0.87; *p* = 0.001) and the ulnar nerve’s SNAP (*r* = −0.87; *p* = 0,002). Positive correlations were found with the assessed items of the MHOQ (*r* = 0.87; *p* = 0.001), skin auto-fluorescence (*r* = 0.90; *p* < 0.001), and HbA1c levels (*r* = 0.66; *p* = 0.040). HbA1c levels showed negative correlations with the total Pegboard score (*r* = −0.68; *p* = 0.031) and with skin auto-fluorescence (*r* = 0.75; *p* = 0.012). For both hsTNT and HbA1c, no correlations were found with age, BMI), or GFR. A detailed summary of all correlations of hsTNT, HbA1c, and FA values with clinical, serological, electrophysiological and demographic data is provided in Table 2 and in Supplementary Table 1.

## Discussion

To our knowledge, this study is the first to assess peripheral nerve DTI at the level of the upper arm in patients with T2D and prediabetes. The results show that the average FA of the nerves at the upper arm is correlated with functional skills at the level of the hands and electrophysiological parameters at the level of the lower arms. This is in accordance with previous studies that found FA to be a reliable correlate for structural nerve integrity (Lehmann et al., 2010; Vaeggemose et al., 2017, 2020; Jende et al., 2021).

Average FA values for the upper arm nerves ranged between 0.40 and 0.46, whereby FA values were increasing from ulnar to median and radial nerves, see Table 1. Kronlage et al. reported FA values of upper limb nerves in a healthy cohort and found an age dependency [Figure 4 in Kronlage et al. (2018)]: mean FA values for ulnar, median, and radial nerve at age 59.4 years (the average age of participants in our study) were 0.51 ± 0.07, 0.55 ± 0.08, 0.57 ± 0.09, respectively. These FA values are larger than the FA’s found for the prediabetes and diabetes patients in our cohort. This can be due to impaired structural nerve integrity, e.g., of the neuropathy patients; yet there were also differences in data acquisition such as a different MRI machine, extremity coil and DTI parameters. In addition, Kronlage et al. (2018) determined FA values for the central four slices, covering a distance of approximately 1.6 cm, while we determined averaged FA values over nerve distances larger than 9.6 cm (24 slices times 4 mm).

Another study by Breitenseher et al. found an average FA value of 0.44 ± 0.04 (range: 0.38–0.51) for averaged ulnar nerve FAs in different positions along the nerve covering 6.4 cm in a healthy collective (20 healthy participants, average age: 40 years), see Figure 3 in Breitenseher et al. (2015). Lowest FA values were found in the region of the cubital tunnel. Correcting for age (from 40 to 59.4 years) as suggested by Kronlage et al. (2018) with a correction factor of −0.0026 × [(age difference)/years] would yield an average FA value of 0.39 ± 0.04 for the Breitenseher cohort, which is in fact slightly lower than the FA value we found, also possibly due to differences in data acquisition.

This study further found that the median, ulnar, and radial nerve’s FA was associated with hsTNT levels. This is in line with previous studies which showed that hsTNT is well associated with microvascular damage and the severity of DN at the level of the lower limbs in patients with T2D (Li et al., 2014; Jende et al., 2020a). The results of this study now indicate that hsTNT also parallels structural nerve damage at the level of the upper limbs.

This study further found a positive correlation for hsTNT and HbA1c levels, and a negative correlation between HbA1c and the average FA of the upper arm nerves. This finding may suggest that hyperglycemia is the primary cause for nerve damage at the level of the upper limbs and that hsTNT is secondarily elevated as a consequence of hyperglycemia-induced microangiopathy. However, the impact of glucose control on the development and progression of DN in patients with T2D is controversially discussed since longitudinal clinical trials found no evidence for a positive impact of glucose control on the progression of DN in patients with T2D (Callaghan et al., 2012). Several studies have found that vascular factors appear to pose a more important risk factor for the development and progression of DN in T2D than glycemic control alone (Tesfaye et al., 2005). Taken together with the results of histological studies that found nerve ischemia to be a contributing factor to DN in T2D (Dyck et al., 1984, 1986a,b), our results and the results of imaging studies mentioned above, it seems well possible that even at early stages of DN or even before the occurrence of clinical symptoms the nerves of the proximal upper extremities show relatively low FA values even though HbA1c levels appear to be relatively normal.

The finding that both average FA and hsTNT were correlated with skin auto-fluorescence, a marker associated with hyperglycaemia, further supports this assumption. It is important to consider, however, that the correlation between FA and HbA1c did not remain significant in a partial correlation analysis controlled for hsTNT, whereas the correlation between hsTNT and FA remained stable after controlling for HbA1c. Therefore, the association between hsTNT and FA appears to be independent of HbA1c, indicating that microangiopathy leading to nerve damage is not solely related to hyperglycemia in the assessed cohort.

One may of course argue that skin auto-fluorescence, hsTNT, and FA are parameters that have been shown to be associated with age and that, therefore, the findings obtained from a small cohort of patients ranging from age 42 to 75 years may only represent the process of aging (Gerrits et al., 2008; Gore et al., 2014; Kronlage et al., 2018). While we cannot exclude an impact of the physiological process of aging on the results obtained, it should be considered that skin auto-fluorescence, FA, and hsTNT showed no correlation with age and that, therefore, patient age does not appear to be the main contributor to decreased FA or elevated hsTNT levels in the cohort examined. One may further argue that hsTNT tends to be elevated in patients with impaired renal function and that, as a consequence, the results only represent an impairment of renal function that has been shown to be associated with a worsening of symptoms in DPN (Wayand et al., 2000). One has to take into account, however, that we found no correlations between GFR and hsTNT. Also, no correlation was found between GFR and FA.

Our study has several limitations: first, the size of our cohort does not allow excluding all potential confounders or the assessment of gender differences. Also, we did not examine a control group to look for differences of FA or hsTNT levels. It should be considered, however, that the primary aim of this study was to investigate whether the FA of the proximal nerves at the upper limb is associated with clinical and serological parameters in patients with elevated blood glucose levels that are comparable to those found for the FA of the sciatic nerve.

Moreover, the small sample size of our study does not allow a meaningful statistical assessment of differences between patients with and without DN. While this is a limitation, it should be considered that the development of DN has been shown to be a continuous process during which subclinical nerve damage can already be visualized and quantified with MRN imaging prior to the occurrence of clinical symptoms (Groener et al., 2019; Morgenstern et al., 2021). In fact, the objective of this study was not to compare patients with and without DN, but to assess whether hsTNT is associated with a decrease in FA, that represents neurostructural deterioration mainly related to demyelination, and functional loss of proximal nerves at the upper limbs in patients with impaired glucose control. Our results indicate that an increase in hsTNT is associated with a decrease in FA, which, once the FA is low enough, causes an impairment of nerve function that ultimately results in neuropathic symptoms.

## Conclusion

In summary, this study found correlations between hsTNT and the FA of the proximal nerves of the upper arm in a group of patients with prediabetes and T2D. This correlation was independent from HbA1c levels. Both hsTNT and FA were associated with functional and electrophysiological assessments of the arms and hands, indicating that microangiopathy contributes to structural nerve damage in prediabetes and T2D. Further longitudinal studies are warranted to assess the diagnostic and prognostic value of hsTNT and the nerves’ FA at the level of the upper arms with regard to the development and progression of diabetic polyneuropathy in prediabetes and T2D.

## Data Availability Statement

The datasets presented in this article are not readily available because they contain sensitive patient information. The data supporting the conclusions of this article will be made available upon reasonable request by any qualified researcher. Requests to access the datasets should be directed to FK, felix.kurz@med.uni-heidelberg.de.

## Ethics Statement

The studies involving human participants were reviewed and approved by the Ethikkommission der Medizinischen Fakultät der Universität Heidelberg, Heidelberg, Germany. The patients/participants provided their written informed consent to participate in this study.

## Author Contributions

JJ, ZK, PN, MB, and FK designed and coordinated the study. JJ, ZK, JM, CM, AJ, and FK contributed to the organization of participants. JJ, AJ, PR, and FK collected MR data. JJ and FK developed image analysis tools, analyzed the data, and wrote the manuscript. ZK, JM, and SK collected the clinical, serological, and electrophysiological data. All authors contributed to the article and approved the submitted version.

## Conflict of Interest

The authors declare that the research was conducted in the absence of any commercial or financial relationships that could be construed as a potential conflict of interest.

## Publisher’s Note

All claims expressed in this article are solely those of the authors and do not necessarily represent those of their affiliated organizations, or those of the publisher, the editors and the reviewers. Any product that may be evaluated in this article, or claim that may be made by its manufacturer, is not guaranteed or endorsed by the publisher.

## References

[B1] Alleman C. J. M. Westerhout K. Y. Hensen M. Chambers C. Stoker M. Long S. (2015). Humanistic and economic burden of painful diabetic peripheral neuropathy in Europe: a review of the literature. *Diabetes Res. Clin. Pract.* 109 215–225. 10.1016/j.diabres.2015.04.031 26008721

[B2] Breitenseher J. B. Kranz G. Hold A. Berzaczy D. Nemec S. F. Sycha T. (2015). MR neurography of ulnar nerve entrapment at the cubital tunnel: a diffusion tensor imaging study. *Eur. Radiol.* 25 1911–1918. 10.1007/s00330-015-3613-7 25680717

[B3] Callaghan B. C. Cheng H. T. Stables C. L. Smith A. L. Feldman E. L. (2012). Diabetic neuropathy: clinical manifestations and current treatments. *Lancet Neurol.* 11 521–534. 10.1016/S1474-4422(12)70065-0 22608666 PMC4254767

[B4] Davies M. Brophy S. Williams R. Taylor A. (2006). The prevalence, severity, and impact of painful diabetic peripheral neuropathy in type 2 diabetes. *Diabetes Care* 29 1518–1522. 10.2337/dc05-2228 16801572

[B5] Dyck P. J. Karnes J. L. Daube J. O’Brien P. Service F. J. (1985). Clinical and neuropathological criteria for the diagnosis and staging of diabetic polyneuropathy. *Brain* 108(Pt 4) 861–880. 10.1093/brain/108.4.861 4075076

[B6] Dyck P. J. Karnes J. L. O’Brien P. Okazaki H. Lais A. Engelstad J. (1986a). The spatial distribution of fiber loss in diabetic polyneuropathy suggests ischemia. *Ann. Neurol.* 19 440–449. 10.1002/ana.410190504 3717907

[B7] Dyck P. J. Lais A. Karnes J. L. Obrien P. Rizza R. (1986b). Fiber Loss Is Primary and Multlfocal in Surd Nerves in Diabetic Polyneuropathy. *Ann. Neurol.* 19 425–439. 3717906 10.1002/ana.410190503

[B8] Dyck P. J. Karnes J. O’Brien P. Nukada H. Lais A. Low P. (1984). Spatial pattern of nerve fiber abnormality indicative of pathologic mechanism. *Am. J. Pathol.* 117 225–238. 6333825 PMC1900442

[B9] Edward R. Abdelalim A. M. Ashour A. S. Afifi L. Al-Athwari A. (2020). A study of diffusion tensor imaging of median nerve in diabetic peripheral neuropathy. *Egypt. J. Neurol. Psychiatr. Neurosurg.* 56:42. 10.1186/s41983-020-00172-5

[B10] Feldman E. L. Nave K.-A. Jensen T. S. Bennett D. L. H. (2017). New horizons in diabetic neuropathy: mechanisms, bioenergetics, and pain. *Neuron* 93 1296–1313. 10.1016/j.neuron.2017.02.005 28334605 PMC5400015

[B11] Gerrits E. G. Lutgers H. L. Kleefstra N. Graaff R. Groenier K. H. Smit A. J. (2008). Skin autofluorescence. *Diabetes Care* 31 517–521. 10.2337/DC07-1755 18039805

[B12] Giannitsis E. Kurz K. Hallermayer K. Jarausch J. Jaffe A. S. Katus H. A. (2010). Analytical validation of a high-sensitivity cardiac troponin T assay. *Clin. Chem.* 56, 254–61. 10.1373/clinchem.2009.132654 19959623

[B13] Gibbons C. H. Freeman R. Veves A. (2010). Diabetic neuropathy. *Diabetes Care* 33 2629–2634. 10.2337/DC10-0763 20805259 PMC2992203

[B14] Gore M. O. Seliger S. L. deFilippi C. R. Nambi V. Christenson R. H. Hashim I. A. (2014). Age- and sex-dependent upper reference limits for the high-sensitivity cardiac Troponin T assay. *J. Am. Coll. Cardiol.* 63 1441–1448. 10.1016/J.JACC.2013.12.032 24530665 PMC3984900

[B15] Groener J. B. Jende J. M. E. Kurz F. T. Kender Z. Treede R.-D. Schuh-Hofer S. (2019). Understanding diabetic neuropathy: from subclinical nerve lesions to severe nerve fiber deficits. a cross-sectional study in patients with type 2 diabetes and healthy controls. *Diabetes* 69 436–447. 10.2337/db19-0197 31826867

[B16] Jende J. M. E. Groener J. B. Kender Z. Hahn A. Morgenstern J. Heiland S. (2020a). Troponin T parallels structural nerve damage in type 2 diabetes A cross sectional study using magnetic resonance neurography. *Diabetes* 69 713–723. 10.2337/db19-1094 31974140

[B17] Jende J. M. E. Groener J. B. Kender Z. Rother C. Hahn A. Hilgenfeld T. (2020b). Structural nerve remodeling on 3-T MR Neurography differs between painful and painless diabetic polyneuropathy in either type 1 or type 2 diabetes. *Radiology* 294 405–414. 10.1148/radiol.2019191347 31891321

[B18] Jende J. M. E. Groener J. B. Oikonomou D. Heiland S. Kopf S. Pham M. (2018). Diabetic neuropathy differs between type 1 and type 2 diabetes: insights from magnetic resonance neurography. *Ann. Neurol.* 83 588–598. 29443416 10.1002/ana.25182

[B19] Jende J. M. E. Kender Z. Mooshage C. Groener J. B. Alvarez-Ramos L. Kollmer J. (2021). Diffusion tensor imaging of the sciatic nerve as a surrogate marker for nerve functionality of the upper and lower limb in patients with diabetes and prediabetes. *Front. Neurosci.* 15:190. 10.3389/fnins.2021.642589 33746707 PMC7966816

[B20] Jeon T. Fung M. M. Koch K. M. Tan E. T. Sneag D. B. (2018). Peripheral nerve diffusion tensor imaging: overview, pitfalls, and future directions. *J. Magn. Reson. Imaging* 47 1171–1189. 10.1002/jmri.25876 29083521

[B21] Kronlage M. Schwehr V. Schwarz D. Godel T. Uhlmann L. Heiland S. (2018). Peripheral nerve diffusion tensor imaging (DTI): normal values and demographic determinants in a cohort of 60 healthy individuals. *Eur. Radiol.* 28 1801–1808. 10.1007/s00330-017-5134-z 29230526

[B22] Lehmann H. C. Zhang J. Mori S. Sheikh K. A. (2010). Diffusion tensor imaging to assess axonal regeneration in peripheral nerves. *Exp. Neurol.* 223 238–244. 10.1016/j.expneurol.2009.10.012 19879260 PMC3038603

[B23] Levey A. S. Stevens L. A. Schmid C. H. Zhang Y. L. Castro A. F. Feldman H. I. (2009). A new equation to estimate glomerular filtration rate. *Ann. Int. Med.* 150 604–612. 19414839 10.7326/0003-4819-150-9-200905050-00006 PMC2763564

[B24] Li Q. Ryan L. Sattar N. Chalmers J. Woodward M. Poulter N. (2014). Do cardiac biomarkers NT-proBNP and hsTnT predict microvascular events in patients with type 2 diabetes? results from the ADVANCE trial. *Diabetes Care* 37 2202–2210. 10.2337/dc13-2625 24879844

[B25] Marmoy O. R. Furlong P. L. Moore C. E. G. (2019). Upper and lower limb motor axons demonstrate differential excitability and accommodation to strong hyperpolarizing currents during induced hyperthermia. *J. Neurophysiol.* 121 2061–2070. 10.1152/jn.00464.2018 30917073 PMC6620697

[B26] Morgenstern J. Groener J. B. Jende J. M. E. Kurz F. T. Strom A. Göpfert J. (2021). Neuron-specific biomarkers predict hypo- and hyperalgesia in individuals with diabetic peripheral neuropathy. *Diabetologia* 64 2843–2855. 10.1007/s00125-021-05557-6 34480211 PMC8563617

[B27] Nobue A. Kunimasa Y. Tsuneishi H. Sano K. Oda H. Ishikawa M. (2020). Limb-Specific features and asymmetry of nerve conduction velocity and nerve trunk size in human. *Front. Physiol.* 11:1576. 10.3389/fphys.2020.609006 33343400 PMC7744784

[B28] O’Donnell L. J. Suter Y. Rigolo L. Kahali P. Zhang F. Norton I. (2017). Automated white matter fiber tract identification in patients with brain tumors. *Neuroimage Clin.* 13 138–153. 10.1016/J.NICL.2016.11.023 27981029 PMC5144756

[B29] Pham M. Oikonomou D. Hornung M. Weiler M. Heiland S. Bäumer P. (2015). Magnetic resonance neurography detects diabetic neuropathy early and with proximal predominance. *Ann. Neurol.* 78 939–948. 26381658 10.1002/ana.24524 PMC5132066

[B30] Shauver M. J. Chung K. C. (2013). The michigan hand outcomes questionnaire (MHQ) after 15 years of field trial. *Plast Reconstr. Surg.* 131 779e–787e. 10.1097/PRS.0b013e3182865d83 23629117 PMC3641688

[B31] Spallone V. Greco C. (2013). Painful and painless diabetic neuropathy: one disease or two? *Curr. Diab. Rep.* 13 533–549. 10.1007/s11892-013-0387-7 23677582

[B32] Tesfaye S. Chaturvedi N. Eaton S. E. M. Ward J. D. Manes C. Ionescu-Tirgoviste C. (2005). Vascular risk factors and diabetic neuropathy. *N. Engl. J. Med.* 352 341–350. 10.1056/NEJMoa032782 15673800

[B33] Tiffin J. Asher E. J. (1948). The purdue pegboard: norms and studies of reliability and validity. *J. Appl. Psychol.* 32 234–247. 10.1037/h0061266 18867059

[B34] Vaeggemose M. Haakma W. Pham M. Ringgaard S. Tankisi H. Ejskjaer N. (2020). Diffusion tensor imaging MR neurography detects polyneuropathy in type 2 diabetes. *J. Diabetes Complications* 34:107439. 10.1016/j.jdiacomp.2019.107439 31672457

[B35] Vaeggemose M. Pham M. Ringgaard S. Tankisi H. Ejskjaer N. Heiland S. (2017). Magnetic resonance neurography visualizes abnormalities in sciatic and tibial nerves in patients with type 1 diabetes and neuropathy. *Diabetes* 66 1779–1788. 10.2337/db16-1049 28432188

[B36] Wayand D. Baum H. Schätzle G. Schärf J. Neumeier D. Rodeheffer R. J. (2000). Cardiac troponin T and I in end-stage renal failure. *Clin. Chem.* 46 1345–1350. 10.1373/clinchem.2009.130740 10973864

[B37] Xia X. Dai L. Zhou H. Chen P. Liu S. Yang W. (2021). Assessment of peripheral neuropathy in type 2 diabetes by diffusion tensor imaging: a case-control study. *Eur. J. Radiol.* 145:110007. 10.1016/j.ejrad.2021.110007 34758418

[B38] Young M. J. Boulton A. J. M. Macleod A. F. Williams D. R. R. Onksen P. H. S. (1993). A multicentre study of the prevalence of diabetic peripheral neuropathy in the United Kingdom hospital clinic population. *Diabetologia* 36 150–154. 8458529 10.1007/BF00400697

