# Supplementary material for: Fractional Anisotropy and Troponin T Parallel Structural Nerve Damage at the Upper Extremities in a Group of Patients With Prediabetes and Type 2 Diabetes – A Study Using 3T Magnetic Resonance Neurography
Source: Front Neurosci. 2022 Jan 24;15:741494. doi: 10.3389/fnins.2021.741494 (PMC8818845; doi:10.3389/fnins.2021.741494)
Supplement: Supplementary file 1 [file Table_1.docx]

**Supplementary Table 1:** Correlation of HbA1c, high sensitivity Troponin T and fractional anisotropy of the nerves of the upper limb with electrophysiological data.

|  | HbA1c | | hsTNT (pg/ml) | | Average FA | | Median nerve FA | | Ulnar nerve FA | | Radial nerve FA | |  |
| --- | --- | --- | --- | --- | --- | --- | --- | --- | --- | --- | --- | --- | --- |
|  | r | p | r | p | r | p | r | p | r | p | r | p | |
| Median nerve motor NCV (m/s) | -0.49 | 0.176 | -0.49 | 0.181 | 0.62 | 0.076 | 0.38 | 0.306 | 0.49 | 0.179 | 0.74 | 0.023 | |
| Median nerve CMAP (µV) | -0.67 | 0.071 | -0.54 | 0.163 | 0.42 | 0.304 | 0.29 | 0.486 | 0.29 | 0.491 | 0.52 | 0.189 | |
| Median nerve DML (ms) | -0.13 | 0.758 | 0.33 | 0.428 | 0.15 | 0.725 | -0.04 | 0.918 | 0.34 | 0.406 | 0.14 | 0.733 | |
| Median nerve sensory NCV (m/s) | -0.18 | 0.674 | -0.21 | 0.619 | -0.25 | 0.544 | -0.26 | 0.534 | -0.70 | 0.055 | 0.04 | 0.918 | |
| Median nerve SNAP (µV) | -0.19 | 0.675 | -0.68 | 0.092 | 0.80 | 0.029 | 0.87 | 0.010 | 0.87 | 0.010 | 0.60 | 0.151 | |
| Ulnar nerve motor NCV (m/s) | -0.53 | 0.142 | -0.57 | 0.107 | 0.29 | 0.441 | 0.22 | 0.578 | -0.05 | 0.896 | 0.49 | 0.176 | |
| Ulnar nerve CMAP (µV) | -0.23 | 0.558 | -0.39 | 0.297 | 0.26 | 0.507 | 0.23 | 0.544 | -0.03 | 0.947 | 0.39 | 0.305 | |
| Ulnar nerve DML (ms) | -0.01 | 0.981 | 0.05 | 0.907 | 0.25 | 0.522 | 0.28 | 0.459 | 0.29 | 0.451 | 0.15 | 0.693 | |
| Ulnar nerve sensory NCV (m/s) | 0.33 | 0.428 | -0.11 | 0.802 | -0.48 | 0.228 | -0.48 | 0.224 | -0.53 | 0.178 | -0.35 | 0.398 | |
| Ulnar nerve SNAP (µV) | -0.42 | 0.264 | -0.87 | 0.002 | 0.89 | 0.001 | 0.90 | 0.001 | 0.74 | 0.022 | 0.80 | 0.010 | |
| NCV = nerve conduction velocity; DML = distal motor latency; CMAP = Compound motor action potential; SNAP = Sensory nerve action potential; µV = microvolts; ms = miliseconds; m/s = meters per second | | | | | | | | | | | | | |
